# Meteorological Table

**Published:** 1811-03

**Authors:** 


### METEOROLOGICAL TABLE.

*From Jan. 28, to Feb. 25.*

| | D | Therm. | | | Barom. | | | Hygrom. dry——damp | | | | Weather. |
|---|---|---|---|---|---|---|---|---|---|---|---|---|
| | 28 | 35 | 34 | 31 | 29$^{6}$ | — | — | | 23 | 8 | 9 | C. . . F. . . . .—W. . |
| | 29 | 28 | 31 | 34 | — | — | —$^{7}$ | | 15 | 6 | 4 | F . . .—. . . . W. .NW. . |
| | 30 | 27 | 31 | 34 | — | — | —$^{5}$ | | 10 | 9 | 3 | F. . .C. . .snow.SE. .S . |
| ◐ | 31 | 35 | 42 | — | —$^{2}$ | —$^{1}$ | — | | 40 | 52 | 65 | C. . . . R. .C. . .SE . S. . . |
| | 1 | 40 | 43 | 42 | —$^{3}$ | — | —$^{5}$ | | 55 | 43 | 49 | P . R . . . C . . .R. .SW. .S. . . |
| | 2 | 42 | 45 | — | —$^{6}$ | —$^{5}$ | —$^{4}$ | | 51 | — | 50 | C. . . . .F. R . SE . . |
| | 3 | 46 | 44 | 40 | —$^{5}$ | —$^{4}$ | —$^{8}$ | | 48 | 43 | 38 | F. . . C . . . F. . . . SW .W. . . |
| | 4 | 38 | — | 42 | 30 | — | —$^{1}$ | | 38 | — | 40 | F . .—SE . . SW . . |
| | 5 | 39 | 40 | 42 | 30 | 29$^{8}$ | —$^{7}$ | | 40 | 39 | — | F. . . C . . . . C. . SE . . SW. |
| | 6 | 45 | 48 | — | —$^{6}$ | —$^{5}$ | —$^{4}$ | | 42 | 40 | 34 | C. . . F. . C. . . S . . SW . |
| | 7 | 46 | 49 | 44 | —$^{6}$ | —$^{7}$ | —$^{8}$ | | 27 | 5 | 16 | F. . R . F . .—. . . .W. |
| ○ | 8 | 45 | 47 | 48 | —$^{7}$ | —$^{5}$ | — | | 25 | 42 | 49 | C . . R . . —W . . . |
| | 9 | 45 | 48 | 47 | —$^{5}$ | — | —$^{8}$ | | 46 | 37 | — | C. . . . F . .—. E. .W. .: |
| | 10 | 47 | 51 | — | — | — | —$^{6}$ | | 45 | 49 | 51 | C. . . R . . C. . . . .SW . |
| | 11 | 50 | 51 | 49 | —$^{5}$ | —$^{4}$ | — | | 54 | 46 | 48 | C . R . . C . . . W . : . |
| | 12 | 48 | — | 44 | —$^{3}$ | —$^{1}$ | —$^{3}$ | | 45 | 46 | 30 | C. . . . R . . F. . . . S. . |
| | 13 | 39 | — | 38 | —$^{3}$ | —$^{2}$ | — | | 35 | 40 | 15 | F. . .s. .h. . .F. . . .W. .NW. . . |
| | 14 | 38 | 42 | 40 | — | —$^{5}$ | —$^{6}$ | | 25 | 11 | 9 | F . —. . .C. . . . W . . . |
| | 15 | 39 | 41 | 39 | —$^{7}$ | —$^{5}$ | —$^{1}$ | — 2 — | 5 | — | 24 | C. . . —R . . . S E. . E. . |
| ◑ | 16 | 39 | — | 35 | —$^{4}$ | —$^{9}$ | 30 | | 31 | 11 | 9 | C. . . F. —. . .NW. . .N. . . |
| | 17 | 33 | 38 | — | —$^{2}$ | — | — | | 8 | 5 | 9 | F. . .Fog . . . .C. . .—N . E. . |
| | 18 | 38 | 43 | 40 | 30 | — | — | — 3 — | 10 | — | 2 | C. . . . F. . . C . .F. . . .S. . . |
| | 19 | 36 | — | 37 | 29$^{3}$ | — | —$^{8}$ | — — 5 | 5 | — | — | F . . —. . . . —SE . . |
| | 20 | 37 | 41 | — | —$^{7}$ | — | —$^{6}$ | | 4 | 5 | 15 | Fog . . . C. . . R . .E . . |
| | 21 | 40 | 45 | — | —$^{5}$ | —$^{2}$ | 29 | | 34 | 22 | 35 | C . . . . R . —. . E. .SE. . |
| | 22 | 44 | 48 | 43 | — | —$^{1}$ | —$^{2}$ | | 40 | 18 | 24 | F . . — . . .—. . . .W. .S . . |
| ● | 23 | 43 | 46 | 44 | —$^{2}$ | — | — | | 25 | 15 | 30 | F. .R. .F. . .R. . in night S . . |
| | 24 | 45 | 49 | 45 | — | —$^{1}$ | 29 | | 36 | 25 | 32 | C. . .R. .—. .C. . S . . SE . . |
| | 25 | 41 | 47 | 44 | —$^{1}$ | —$^{3}$ | —$^{4}$ | | 33 | 4 | 20 | C. . . .F. . .—. . .NW. . .W. . |

30, light fall of snow in the evening; in the night and morning of the 31st, falls in great quantity, lying a foot thick on the level, in the fields; wastes rapidly with rain in the day.

1st, Snow entirely gone.

2d, Very soft and temperate, with light rain.

3d, Stormy wind from the west.

4th, Frosty, fair, and in the evening temperate.

13th, Until this day the weather has been remarkably temperate for the season. Heavy storm of hail mingled with snow from the N.W. Evening clear and stars brilliant, as they were on the evening of the 12th.

This month may be considered, as having been remarkably temperate, the Therm. having once only been as low as 33 for a few hours, and has been as high as 51, and generally between 40 and 50. Snow only in a small quantity, once succeeded by a storm of hail. The month of February is esteemed the wettest of the year. The present has produced but little rain. On 13 days there has been rain, but in small quantity. On 20 days of 25 the wind has been from S. S.W. or S.E.

*Princes Street, Cavendish Square.*

